# Identification of candidate loci regulating seed-associated traits in soybean using genome-wide association study and image-based high-throughput phenotyping

**DOI:** 10.3389/fpls.2026.1727442

**Published:** 2026-03-02

**Authors:** Sreeparna Chowdhury, Byeong Hee Kang, Seo-Young Shin, Won-Ho Lee, Da-Yeon Kim, Jeong-Ho Baek, Seong-Hoon Kim, Bo-Keun Ha

**Affiliations:** 1 Department of Applied Plant Science, Chonnam National University, Gwangju, Republic of Korea; 2 BK21 Interdisciplinary Program in IT-Bio Convergence System, Chonnam National University, Gwangju, Republic of Korea; 3 Department of Agricultural Biotechnology, National Institute of Agricultural Sciences, Rural Development Administration, Jeonju, Republic of Korea; 4 National Agrobiodiversity Center, National Institute of Agricultural Sciences, Rural Development Administration, Jeonju, Republic of Korea

**Keywords:** candidate gene, genome-wide association study, haplotype analysis, high-throughput phenotyping (HTP), QTL, seed trait, soybean

## Abstract

Seed-associated traits such as flowering, maturity, and seed size are critical determinants of yield and seed morphology in soybean. However, their reliable evaluation is often hindered by polygenic control and strong environmental influences. In this study, we performed a genome-wide association study (GWAS) across 374 soybean accessions evaluated under field conditions during 2020 and 2021 growing seasons in Rural Development Administration, Jeonju, South Korea. The genetic architecture of eight traits was investigated including two reproductive traits: days to flowering (DtF) and days to maturity (DtM), and six seed size-related traits: seed length (SL), seed width (SW), seed thickness (ST), seed area (SA), seed volume (SV), and 100-seed weight (HSW). Among these, SL, SW, ST, SA, and SV were measured using an image-based high-throughput phenotyping platform. Correlation analysis revealed strong associations within reproductive traits and within seed size traits, with relatively weak correlations between reproductive and seed size traits (r ≤ 0.45) suggesting largely independent genetic regulation. GWAS identified six significant pleiotropic SNPs: one on chromosome 19 for DtF and DtM, and five on chromosomes 4, 13, and 20 for seed size traits (SL, SW, ST, SV, and HSW). The linkage disequilibrium interval (± 153 kbp) was defined as the stable QTL region for candidate gene mining. One novel QTL, *qSS13* was also identified for seed size. Haplotype analysis revealed three alleles at *qSS4.2* (Hap4B_1, Hap4B_2, Hap4B_3) and two at *qSS13* (Hap13_1, Hap13_2), each regulating SV and HSW. Within the six QTLs, 23 putative genes were identified, including 16 specifically associated with seed size traits. Among these, six genes showed distinct expression patterns between large and small-seeded accessions, suggesting their roles as strong candidates for seed size regulation. Collectively, these findings provide new insights into the genetic control of seed size and offer valuable targets for marker-assisted selection and molecular breeding to improve soybean yield.

## 1 Introduction

Soybean (*Glycine max* [L.] Merr.) is a globally important legume crop, contributing to protein consumption and food security. It is cultivated worldwide for multipurpose uses, such as human consumption, animal feed, edible oil production, and industrial applications, mainly due to its high protein content (~40%) and oil content (~20%) (Tukamuhabwa et al., 2012). In addition to its nutritional value, the size and shape of soybean seeds also considerably influence the market value and consumer preferences (Kumar et al., 2022). However, soybean yield remains two to three times lower than that of other major staple crops (Jiang et al., 2023). According to current statistics (OECD-FAO Agricultural Outlook 2024-2033), China, Indonesia, the European Union, and Sub-Saharan African countries face persistent challenges in stabilizing the soybean supply-demand chain, largely due to insufficient domestic production. This has amplified the global need to improve soybean production.

Historically, domestication and breeding efforts in soybean have primarily focused on improving agronomic traits associated with yield and quality. While advanced breeding technologies can effectively help mitigate global yield-related issues, our understanding of the genes that control these traits remains limited (Karikari et al., 2020). Soybean yield is a complex quantitative trait governed by various factors such as flowering time, maturity duration, pod number, seeds per pod, and seed size and weight (Bhat et al., 2025). Of these factors, seed size and shape components play critical roles in yield, yet their genetic and molecular mechanisms are still largely unknown, hindering yield improvement efforts (Shao et al., 2022). This knowledge gap has prompted researchers to focus on studying seed traits at the genetic and molecular levels (Zhao et al., 2016).

Seed-related traits, such as thickness, length, width, and 100-seed weight, are polygenic and significantly influenced by environmental conditions and genotype-by-environment interactions (Tao et al., 2017). Conventionally, these traits are assessed manually using calipers, which is labor-intensive and tedious for large-scale analyses. However, recent advances in high-throughput phenotyping have greatly improved the efficiency and accuracy of trait measurement. Several studies have utilized image-based phenotyping platforms to evaluate various soybean seed-related traits including seed length, width, thickness, roundness, area, and perimeter (Kim et al., 2022). For instance, one study (Baek et al., 2020) utilized hyper-spectral images to assess seed viability and later developed software for analyzing seed shape and size in soybean. The use of digital imaging techniques now allows for the generation of large-scale quantitative data, facilitating their integration into molecular analyses such as QTL mapping, genome-wide association studies (GWAS), and marker-assisted selection (Ali et al., 2020).

GWAS has emerged as a powerful approach for elucidating and identifying QTLs and candidate genes associated with yield-related traits (Zatybekov et al., 2017). By leveraging historical recombination events and linkage disequilibrium (LD) patterns in diverse populations, GWAS enables precise identification of trait-associated loci. It has been effectively employed to detect QTLs/genes in several staple crops such as rice, maize, wheat, mung bean, and soybean (Ahmed et al., 2024; Han et al., 2024). High-throughput genotyping technologies such as specific-locus amplified fragment sequencing (SLAF-seq), restriction site-associated DNA sequencing (RAD-seq), and genotyping by sequencing (GBS) have enabled the discovery of thousands of SNPs associated with important traits. Previously, the Illumina SoySNP6K iSelect Bead Chip was used to genotype 91 soybean germplasms across maturity groups (MGs), resulting in the detection of 87 SNPs associated with flowering and maturity (Mao et al., 2017). In South Korea, millions of high-quality SNPs from the resequencing of 47 soybean accessions (31 Chinese and 16 Korean) were used to develop a 180K Axiom^®^ Soya SNP array, which has recently been extensively exploited to study various association and NGS analysis (Kim et al., 2023a).

Despite these advances, the functional validation of the QTLs associated with seed traits remains incomplete. Although over 300 QTLs related to seed traits are listed in SoyBase, most have not been functionally validated, limiting their practical use in marker-assisted selection for seed-size improvement. Numerous studies have focused on QTL discovery and marker development for soybean seed size and shape, however, only a few candidate genes have been detected so far (Sun et al., 2021). Some of these, such as the *GmCYP78A* family (Du et al., 2017), *GmCWI* (Tang et al., 2017), and *BIG SEEDS-1* (Ge et al., 2016), have orthologous functions conserved across species, indicating their potential role in seed size regulation. A notable example is the QTL *qSS20–1* on chromosome 20, which has been reported to regulate soybean seed size (Wang et al., 2024). On chromosome 16, a candidate gene SoyZH13_16G122400, located in the qHSW-16 region, exhibited relatively higher expression during seed expansion in large-seeded varieties and is associated with seed weight in soybeans (Zhang et al., 2021). Additionally, *CONSTANS* (*CO*), a key regulator in the photoperiodic pathway, influences the expression of the transcription factor *APETALA2* (*AP2*), thereby affecting seed size and productivity in soybeans (Zhang et al., 2024).

Understanding the genetic architecture of days to flowering (DtF) and maturity (DtM) is also critical for enhancing soybean adaptability and yield (Zhang et al., 2015). Nine significant loci (E1–E8 and J) have been identified as regulators of DtF and DtM in soybean (Watanabe et al., 2012). Five candidate genes (*Glyma.05G101800*, *Glyma.11G140100*, *Glyma.11G142900*, *Glyma.19G099700*, and *Glyma.19G100900*) have been reported to share homology with *Arabidopsis* genes controlling flowering time (Li et al., 2019). These findings collectively illustrate the complex genetic interplay underlying flowering, maturity, and seed traits in soybean, with GWAS providing greater precision in identifying significant candidate genes compared to conventional approaches.

In this study, a genome-wide association approach was employed to dissect the genetic basis of eight seed-associated traits: days to flowering (DtF), days to maturity (DtM), seed length (SL), seed width (SW), seed thickness (ST), seed area (SA), seed volume (SV), and 100-seed weight (HSW). While image-based phenotyping has been increasingly adopted in soybean GWAS, this study advances previous efforts by integrating high-throughput, digital image-based phenotyping platform to quantify multiple seed morphology traits with multi-trait GWAS, haplotype-based dissection, and gene expression profiling in contrasting seed size accessions. The objective of this integrative framework was to identify stable pleiotropic loci, uncover novel seed size QTLs, and breeding relevant haplotypes. Thereby, providing deeper biological insights into the genetic regulation of seed size and to establish a foundation of knowledge to support molecular breeding strategies for improving soybean yield.

## 2 Materials and methods

### 2.1 Experimental materials

A panel of 374 soybean accessions from diverse origins (South Korea, North Korea, China and Japan) was cultivated during the summer and autumn (June to November) of 2020 and 2021 at the experimental station of the Rural Development Administration, Jeonju, South Korea (**Supplementary Table 7**). Each accession was planted in a two-row plot layout consisting 12 hills (single plant per hill) per row, with hills spaced 0.30 m apart within rows and 0.30m within rows. Adjacent accessions plots were spaced 1.5m to minimize inter-plot competition and the experiment was conducted with two replications. After harvest, all mature seeds were dried to a moisture content of 8–12% and stored for further seed trait evaluations.

### 2.2 Phenotypic data collection and evaluation

**Figure 1:**
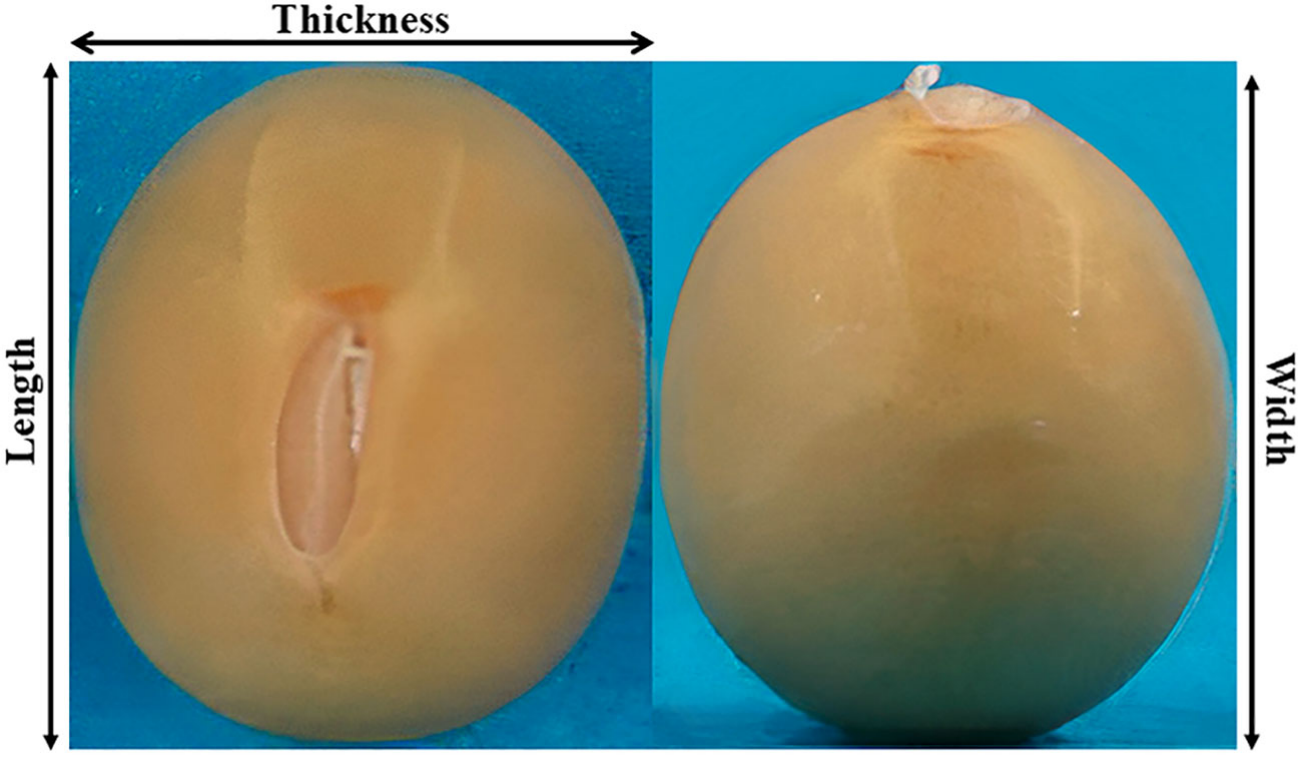
Top and side views of soybean seed morphology captured through digital phenotyping.

The study investigated two reproductive traits, DtF and DtM, as well as six seed-size traits *viz.* SL, SW, ST, SA, SV, and HSW. DtF and DtM were calculated based on the number of days from sowing until 50% of the plants within each plot (24 plants per accession) reached the specific stage of flowering and maturity, respectively. For measuring HSW, mature seeds of each sample were oven-dried at 30 °C for 72 hours, and randomly selected 100 healthy seeds were weighed on an electronic balance in three replicates to obtain the average HSW value in grams. The other seed-size traits were examined using high-throughput seed phenotyping (digital imaging) with 20 seeds per accession. Phenotyping was performed by taking images with a lens (SEL30M35 E30 mm F3.5 Macro; Sony, Tokyo, Japan) fixated on the camera (α-6000; Sony); additionally, CN-T96 light (ProDean; 832 lux) and VILTRONX light (VL-D85T; 2521 lux) were strategically positioned to reduce shadow casting for precise photographing. Top and side view images of each accession were processed and analyzed using ImageJ-based soybean seed phenotyping macros (Ferreira and Rasband, 2012; Schneider et al., 2012; Baek et al., 2020). The image-derived measurements were automatically extracted and saved in CSV format as phenotypic data. Traits like SL, SW, and ST were considered as shown in **Figure 1**. SV was obtained using the formula (Kumar et al., 2013) as in Equation 1:

(1) $$Volume\;=\;\frac{4}{3}\;\pi \;\times \;Length\;\times \;Width\;\times \;Thickness$$

For statistical analysis, the variance components were estimated using an ANOVA-based random effect model, and the phenotypic observation was described using the following statistical model model (Equation 2):

(2) $$Y_{ij}=\mu +G_{i}+E_{j}+\epsilon_{ij}$$

where *Y_ij_* is the trait value of the genotype *i* in replication *j* (year); *μ* as overall mean; *G_i_*was considered as a random effect of genotype, *E_j_* as the environmental (year-wise) replication reflecting variation among the independent cultivation trials, and *ϵ_ij_* represented as residual error. Hence, the broad-sense heritability (*H^2^*) was estimated across years (**Table 1**) using below formula (Equation 3), where *σ^G^* and *σ^E^* denotes the genotypic variance and the environmental variance (year-wise), respectively.

**Table 1:** Descriptive overview of seed-associated traits in 374 soybean accessions.

Trait	Year	Range	Mean ± SE	SD	CV%	Kurtosis	Skewness	*H^2^*
DtF	2020	34–77	47.46 ± 0.40	7.73	16.29	0.67	0.56	0.59
	2021	28–66	38.82 ± 0.37	7.08	18.24	0.11	0.74	
	Mean	31–71.5	43.25 ± 0.35	6.61	15.32	0.97	0.69	
DtM	2020	87–136	110.62 ± 0.49	9.45	8.54	0.08	0.65	0.68
	2021	82–145	108.24 ± 0.76	14.38	14.46	–1.07	0.25	
	Mean	87–137	109.43 ± 0.58	11.19	10.22	–0.63	0.46	
SL	2020	5.81–11.78	8.59 ± 0.06	1.11	12.87	0.43	0.5	0.91
	2021	6.50–12.60	9.07 ± 0.06	1.18	12.97	0.12	0.43	
	Mean	6.42–12.07	8.83 ± 0.06	1.1	12.46	0.37	0.52	
SW	2020	6.67–14.96	10.87 ± 0.08	1.52	13.96	–0.41	0.36	0.72
	2021	7.01–15.94	11.48 ± 0.08	1.5	13.02	–0.06	–0.15	
	Mean	7.54–14.48	11.18 ± 0.07	1.33	11.93	–0.23	0.15	
ST	2020	3.91–8.67	6.26 ± 0.04	0.81	13.02	0.08	–0.02	0.92
	2021	3.67–9.67	6.68 ± 0.05	0.95	14.19	0.52	–0.07	
	Mean	3.98–9.17	6.47 ± 0.04	0.86	13.25	0.37	–0.07	
SA	2020	19.39–77.20	42.60 ± 0.52	9.97	23.4	0.25	0.49	0.90
	2021	18.52–87.29	47.77 ± 0.63	12.01	25.13	0.25	0.51	
	Mean	20.93–82.08	45.18 ± 0.56	10.63	23.53	0.26	0.5	
SV	2020	712.75–6221.98	2545.83 ± 49.76	951.9	37.39	0.75	0.89	0.90
	2021	709.22–6573.64	3038.01 ± 58.26	1115	36.69	0.27	0.66	
	Mean	883.23–6397.81	2791.92 ± 42.33	1001	35.86	0.44	0.76	
HSW	2020	6.55–46.50	18.81 ± 0.36	6.97	37.06	0.83	0.88	0.89
	2021	5.63–53.93	24.67 ± 0.50	9.6	38.9	0.32	0.81	
	Mean	6.29–50.22	21.74 ± 0.42	8.1	37.25	0.47	0.83	

*SE, Standard Error; SD, Standard Deviation; CV, Coefficient of Variation; ^H2^, Broad-sense heritability; DtF, Days to Flowering; DtM, Days to Maturity; SL, Seed Length; ST, Seed Thickness; SW, Seed Width; SA, Seed Area; SV, Seed Volume; HSW, 100-Seed Weight; Units for DtF and DtM are days; Units for SL, SE, and ST are mm; Units for SA, SV, and HSW are mm^2^, mm^3^, and g, respectively.

(3) $$H^{2}=\sigma^{G}/\left(\sigma^{G}+\sigma^{E}\right)$$

Descriptive statistical analysis for all traits was carried out in Microsoft Excel 2019. Correlation analysis and broad-sense heritability (*H^2^*) were performed using the R packages *‘PerformanceAnalytics’* (Peterson et al., 2018) and *‘Variability’* (Popat et al., 2020), respectively.

### 2.3 Population structure, linkage disequilibrium, and genome-wide association analysis

The 180K Axiom^®^ Soya SNP array (Lee et al., 2015) was utilized to genotype a set of soybean accessions. SNP callout, population structure, and linkage disequilibrium (LD) estimation were performed as described in a previous study (Kim et al., 2023a). A total of 180,375 SNPs from genotyping data were filtered through imputation, applying a minor allele frequency (MAF)< 0.05, 1000 burn-in and iterations settings, resulting in a reduced set of 84,391 high-quality SNPs for downstream analysis. The population structure and admixture plot (*k* = 3) were modified using QTLmaxV4 (QTLmax Global, Texas, USA), while the average LD decay (r^2^) graph was constructed in TASSEL 5.2.95 (Buckler Lab, Institute for Genomic Diversity, Cornell University) with a default window size of 50 SNPs and the open-source software RStudio v2024.09.1 + 394 (Posit Software, Boston, MA, USA). The LD decay rate is the physical distance where the average pairwise declines to 50% of its highest value (Huang et al., 2010). GWAS for all traits was conducted on the QTLmaxV4 pipeline, employing a mixed linear model (MLM) with default parameters; it incorporates population structure as a fixed effect (Q matrix) and kinship as a random effect (K matrix) to account for confounding due to relatedness and structure, thereby minimizing Type I error in SNP-trait associations. Furthermore, association analysis was conducted using merged phenotype data to enhance resolution across environments (Kim et al., 2019). The Bonferroni method, estimated as p = α/n was followed to estimate the −log_10_(P) threshold for Manhattan and QQ plots, where α = 1 and n = 84,391 SNPs were employed to get the Bonferroni corrected thresholds with corresponding −log_10_(P) values of ≥ 4.9 as the significance threshold.

### 2.4 Haplotype based trait-association analysis

The single-allele effect and multi-allelic effect of significant SNPs were assessed to understand positive alleles for different traits. To generate haplotypes (specifically set multi-alleles) for significant SNPs, the LD between SNP pairs were predicted using Haploview 4.2 (Barrett et al., 2005). The nearest adjacent SNPs flanking within a physical distance of ±153 kbp around significant SNPs were considered haplotype blocks, which were defined based on the “confidence intervals” algorithm (Gabriel et al., 2002). Accessions were classified based on their haplotype alleles within each defined block. Specifically, only the haplotypes with a population frequency ≥10% were included in the statistical analysis to avoid unreliable estimates from rare haplotypes. Subsequently, haplotype–trait associations were evaluated using environment-averaged phenotypic data. The haplotypes were named based on chromosome number, significant SNP order, and haplotype number. For example, in Hap4A_1, ‘4’ indicates chromosome number, ‘A’ represents significant SNP order on the same chromosome, and ‘1’ is the haplotype number.

### 2.5 Candidate gene mining and functional elucidation

Based on significant SNPs identified from GWAS signals, the optimal physical interval for exploring potential candidate genes were defined as ±153 kbp, corresponding to the average LD decay distance observed in the population. All genes within the LD region around the significant and overlapping SNPs were mined from the reference genome model of Williams82 (Wm82.a2.v2) in SoyBase (https://www.soybase.org/) in order to identify the genes and their respective genomic regions, verify their functional annotations related to seed development, seed expansion, flowering, maturity, hormonal signaling, and examine their tissue expressions based on publicly available RNA-seq database (**Supplementary Figure 2**; **Supplementary Tables 5**, **6**). The selection of potential candidate genes was based on trait-associated annotations in QTL regions and a review of literature on model crops. However, the candidate genes were prioritized and recommended based on gene expression analysis.

### 2.6 RNA extraction and qRT-PCR

The qRT-PCR was performed on two contrasting groups of soybean accessions, comprising two small-seeded (IT158022 and IT224885) and two large-seeded (IT185237 and IT185304) (**Supplementary Table 1**) to elucidate the expression patterns of potential candidate genes. The soybean accessions were planted at the Experimental Station of Chonnam National University, Gwangju Province, Republic of Korea in 2024. Fresh seeds were sampled at the R6 stage (full stage) of seed development with three biological replicates. Total RNA extraction was carried out using the Plant RNA Extraction Kit (TaKaRa MiniBEST, Catalog no. 9769; Japan) according to the provided protocol. Synthesis of complementary DNA (cDNA) was accomplished using the LaboPass cDNA Synthesis Kit (Comso Gene Tech, South Korea). Gene-specific primers (**Supplementary Table 2**) were designed using NCBI tools (https://www.ncbi.nlm.nih.gov/tools/primer-blast/) and synthesized by Macrogen (South Korea). *GmActin11* (*Glyma.18g290800*) was included as the internal control gene (housekeeping gene). All qRT-PCR reactions were conducted using Power SYBR Green Master Mix and the ABI Step One Real Time PCR system (Thermo Fisher Scientific, Waltham, MA, USA). Each sample for qRT-PCR analysis had three biological replicates and two technical replicates. The amplification process included incubations at 95 °C for 10 min, followed by cycles of 95 °C for 15 s and 60 °C for 1 min. The 2^−ΔΔCt^ method (Livak and Schmittgen, 2001) was applied to normalize and estimate the relative fold differences in gene expression. We used IT158022 (small-sized seed) accession as a control for ΔΔCt normalization comparing gene expression levels among the selected soybean accessions, while IT224885 was included to capture within-group biological variation among small-seeded accessions.

### 2.7 Multiple comparison and statistical assessment

For qRT-PCR analysis, two technical replicates were averaged for each biological replicate, and statistical comparison among accessions were assessed based on three independent biological replicates per accession. One-way ANOVA followed by Tukey’s Honest Significant Difference (HSD) test was applied to estimate differences in gene expression among four soybean accessions. While Welch’s t-test and Welch’s ANOVA followed by Gomes-Howell employed to assess statistically significant differences among the haplotype groups. All evaluations and visualizations were performed using the IBM SPSS Statistics 29.0.2.0 (IBM Corp., Armonk, New York, USA) software and the RStudio v2024.09.1 + 394 (Posit Software, Boston, MA, USA) statistical environment.

## 3 Results

### 3.1 Phenotypic analysis of soybean seed-associated traits

This study evaluated 374 soybean accessions over two consecutive years (2020-2021). Phenotypic data were recorded for eight seed-associated traits, namely DtF, DtM, SL, SW, ST, SA, SV, and HSW. A wide distribution of these traits was observed among the accessions, indicating considerable variability within the population. Across the two years, the ranges for DtF and DtM were 28–77 days and 82–145 days, respectively. Significant variation was also observed for all seed size traits, with SA ranging from 18.52 to 87.29 mm^2^, SV from 709.22 to 6573.64 mm^3^, and HSW from 5.63 to 53.93 g. The average coefficient of variation (CV) across all traits ranged from 10.22% for DtM to 37.25% for HSW, indicating broad variability within the accessions. Broad-sense heritability (*H²*) was estimated using averaged phenotypic data across year 2020 and 2021. Reproductive traits such as DtF and DtM exhibited comparatively lower *H²* than seed size traits, suggesting that the former are more strongly influenced by environmental factors. A detailed summary of phenotypic statistics for all seed-associated traits is presented in **Table 1**.

**Figure 2:**
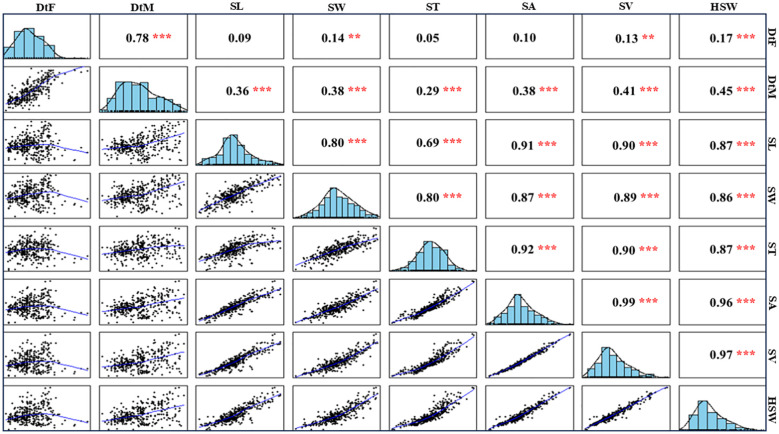
Pearson correlation matrix of seed traits based on the mean phenotypic data from 2020 and 2021. The traits are represented as follows: DtF, Days to Flowering; DtM, Days to Maturity; SL, Seed Length; ST, Seed Thickness; SW, Seed Width; SA, Seed Area; SV, Seed Volume; HSW, 100-Seed Weight. Level of significance is denoted as ***, **, * for p< 0.001,< 0.01,< 0.05, respectively.

Remarkably, correlation analysis of the eight traits revealed a significant positive correlation between reproductive traits (DtF and DtM; *r* = 0.78) and among seed size traits (SL, SW, ST, SA, SV, and HSW; *r* = 0.69–0.99). However, correlations between the two groups (reproductive and seed size traits) were weak and non-significant. The correlation matrix for seed-associated traits is shown in **Figure 2**.

### 3.2 Population structure and linkage disequilibrium

**Figure 3:**
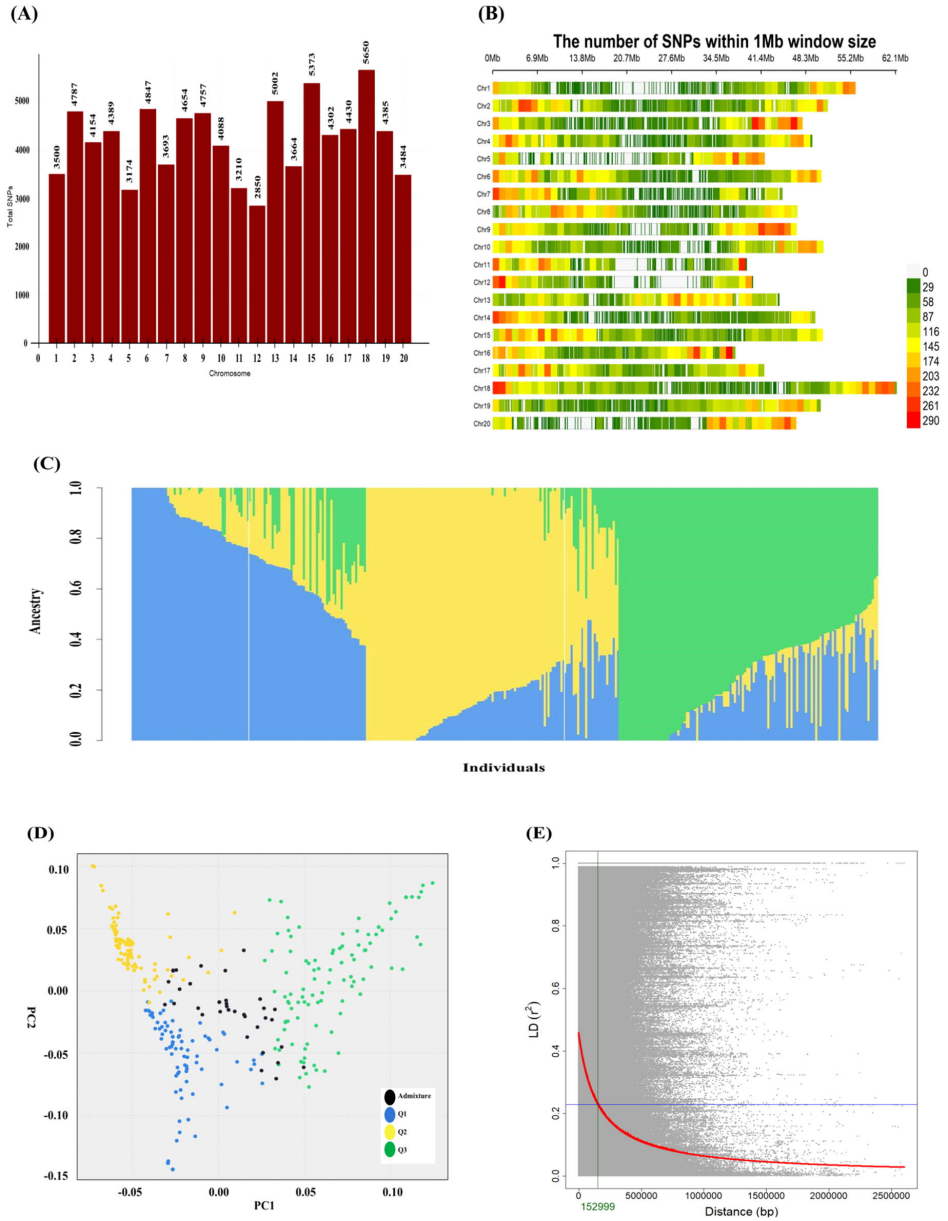
SNP distribution, population structure, and linkage disequilibrium across 374 accessions. **(A)** Genome-wide distribution of 84,391 SNPs across 20 soybean chromosomes. **(B)** SNP density across all chromosomes within 1-Mb windows. Chromosome numbers and lengths are represented on the vertical and top horizontal axes, respectively, and SNP density is illustrated by a color gradient from white to red. **(C)** Admixture plot representing the genetic relationships among soybean accessions, with each color indicating a distinct ancestral lineage. **(D)** PCA plot grouping the accessions into three distinct clusters, with admixture patterns showing common ancestry from two or more clusters. **(E)** LD decay plot across all soybean accessions. The red regression curve illustrates the LD decay rate, while the blue horizontal and green vertical lines over the LD curve indicate the half-decay point (*r^2^* = 0.22) and the corresponding physical distance, respectively.

A total of 84,391 high-quality filtered SNPs were used for genetic analyses, including population structure and LD assessment. These SNPs were distributed throughout the soybean genome, with the highest number (5,650 SNPs) located on chromosome 18 and the lowest (2,850 SNPs) on chromosome 12. The SNP density per 1 Mb window varied, with a maximum of 89.68 SNPs/Mb on chromosome 18 and a minimum of 45.24 SNPs/Mb on chromosome 12 (**Figures 3A, B**). All 84,391 SNPs across the genome were utilized to estimate the kinship matrix and assess population structure. K-means clustering based Bayesian Information Criterion (BIC) revealed clear inflection point at K = 3, indicating the presence of three genetic subgroups in the GWAS panel (**Supplementary Figure 1**). It further supported by admixture analysis, which consistently grouped the accessions into three genetically discrete clusters. And the PCA analysis, revealing first two principal components separate the accessions into three distinct clusters supporting the genetic relatedness among the accessions as illustrated in **Figures 3C, D**. The LD decay curve indicated an initial *r^2^* value of 0.45, which reached half decay rate (*r^2^* = 0.22) at a physical distance of around 153 kbp (**Figure 3E**). Accordingly, the flanking interval of ±153 kbp was designated as the QTL region surrounding significant SNPs detected across multiple years and traits.

### 3.3 Identification of significant SNPs through GWAS

**Figure 4:**
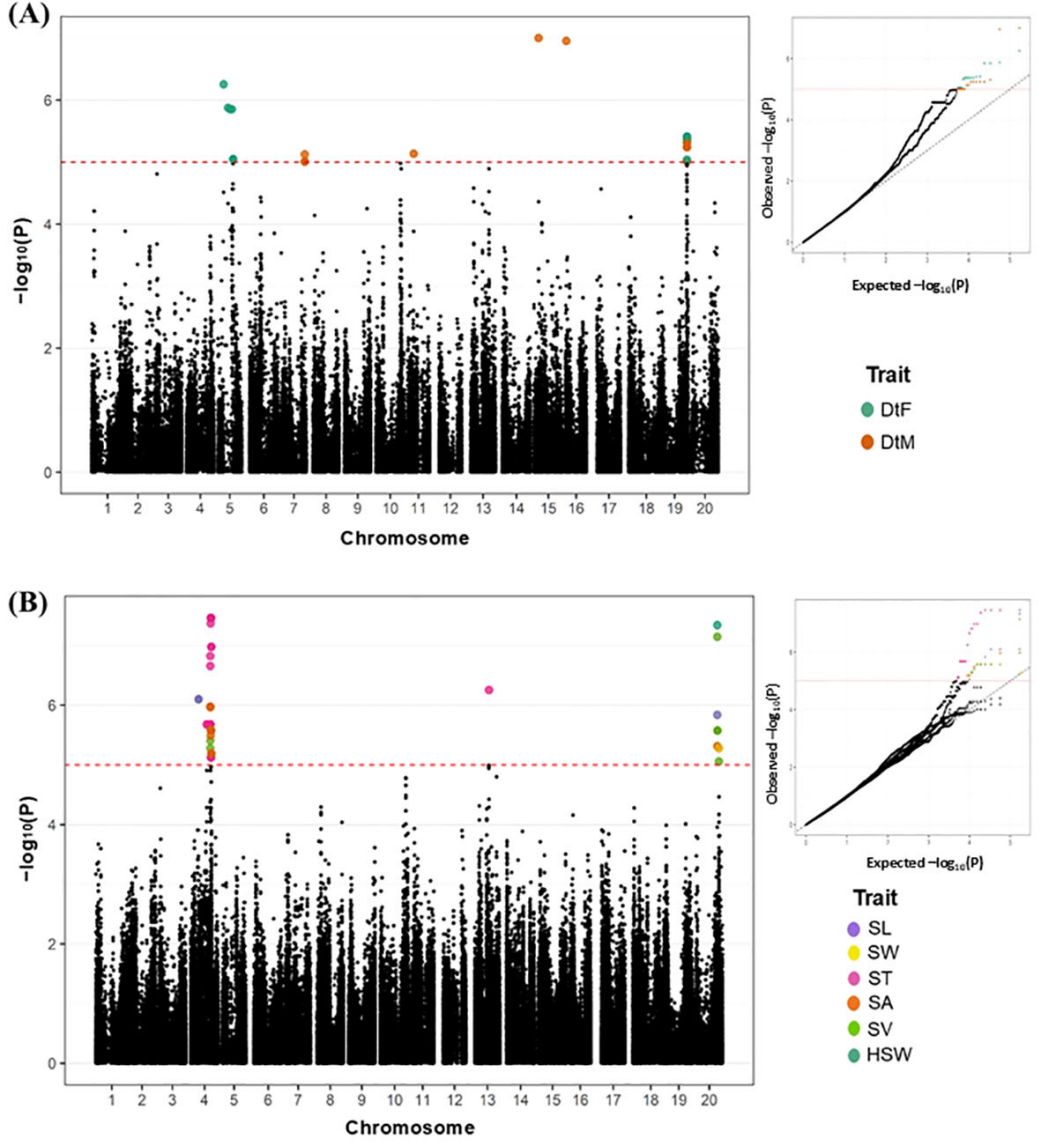
Multi-trait GWAS analysis for seed-associated traits. **(A)** Manhattan and QQ plots for two reproductive traits. **(B)** Manhattan and QQ plots for seed shape- and size-related traits. Manhattan plots highlight SNPs exceeding the Bonferroni threshold, with each color representing a different trait. DtF, Days to Flowering; DtM, Days to Maturity; SL, Seed Length; ST, Seed Thickness; SW, Seed Width; SA, Seed Area; SV, Seed Volume; HSW, 100-Seed Weight.

In total, 99 SNPs showing significant GWAS signals associated with eight traits were detected (**Figure 4**; **Supplementary Table 3**). Among these, only six peak and common SNP signals across traits were identified as stable and significant for QTL detection (**Table 2**). These SNPs were distributed across four chromosomes (Chr. 4, Chr. 13, Chr. 19, and Chr. 20). For reproductive traits (DtF and DtM), one overlapping SNP was identified on Chr. 19 (AX-90418443). For seed size-related traits, five SNPs were detected: two on Chr. 4 (AX-90372240 and AX-90355593), one on Chr. 13 (AX-90423207), and two on Chr. 20 (AX-90393066 and AX-90368935). Since these SNPs were consistently detected across multiple traits, they are considered reliable and potentially influential for seed-related traits, and thus were designated as major pleiotropic QTLs. Accordingly, the QTLs were named following the convention *qSS4.1*, where *“q”* represents a significant SNP, *“SS”* or *“RP”* indicates seed size or reproductive traits, and the number denotes the chromosome and positional order.

**Table 2:** Significant pleiotropic SNPs identified across multiple phenotypic traits.

Trait	SNP	QTL	Chr^a^	BP	−log_10_(P)	MAF^b^
DtF, DtM	AX-90418443	*qRP.19*	19	47,544,809	5.41	0.36
ST, SA, HSW	AX-90372240	*qSS4.1*	4	35,618,477	7.37	0.42
ST, SA, SV, HSW	AX-90355593	*qSS4.2*	4	36,023,075	7.46	0.43
ST, HSW	AX-90423207	*qSS13*	13	24,902,420	6.25	0.13
SL, SA, SV, HSW	AX-90393066	*qSS20.1*	20	37,640,390	7.34	0.08
SW, SV	AX-90368935	*qSS20.2*	20	40,392,430	5.27	0.13

a Chromosome.

b Minor allele frequency; DtF, Days to Flowering; DtM, Days to Maturity; SL, Seed Length; ST, Seed Thickness; SW, Seed Width; SA, Seed Area; SV, Seed Volume; HSW, 100-Seed Weight.

### 3.4 Allelic effects of SNP markers associated with major QTLs for seed traits

The single-allelic effect of selected major QTLs was estimated to evaluate the statistical association and direction of the SNP effect on their respective phenotypic traits (**Table 3**). The allelic effect demonstrates the mean difference between the reference and alternate allele. Hence, a negative allelic effect indicates that the reference allele contributes negatively to the trait and vice versa. For the reproductive traits DtF and DtM, the QTL *qRP19* was governed by the reference allele (GG) and alternate allele (AA). The presence of allele A increased the time period for DtF and DtM, hence conferring a negative effect. For seed traits, *qSS4.1* and *qSS4.2* showed that the reference allele (AA) exhibited a positive effect compared to the alternate allele C and G, respectively. Conversely, the alternate allele (CC) in *qSS13* significantly enhanced the ST and HSW by 16.50% and 44.15%, respectively, compared with the reference allele (TT). Both QTLs on Chr. 20 (*qSS20.1* and *qSS20.2*) revealed that the presence of alternate alleles (AA) increased seed size and shape, thereby contributing positively to trait variation.

**Table 3:** Single allelic effect at significant QTLs detected in the GWAS population.

QTL	Trait	Allele		No. of Samples		Mean		Allelic effect
		Ref	Alt	Ref	Alt	Ref	Alt	
*qRP19*	DtF	G	A	200	144	40.75	46.84	–6.08
	DtM					104.76	116.47	–11.71
*qSS4.1*	ST	A	C	207	149	6.91	6.00	0.91
	SA					50.31	38.76	11.55
	HSW					25.49	17.04	8.45
*qSS4.2*	ST	A	G	203	153	6.93	6.01	0.92
	SA					50.51	38.79	11.71
	SV					3289.53	2198.42	1091.11
	HSW					25.63	17.08	8.54
*qSS13*	ST	T	C	311	45	6.40	7.45	–1.06
	HSW					20.79	29.98	–9.181
*qSS20.1*	SL	G	A	326	29	8.62	10.83	–2.21
	SA					43.99	61.89	–17.90
	SV					2668.00	4507.52	–1839.52
	HSW					20.75	35.61	–14.85
*qSS20.2*	SW	C	A	310	45	10.97	12.86	–1.89
	SV					2636.87	4067.92	–1431.05

*Ref, Reference allele; Alt, Alternate allele; SL, SW, ST are expressed in mm, SA in mm^2^, SV in mm^3^, and HSW in g. DtF, Days to Flowering; DtM, Days to Maturity; SL, Seed Length; ST, Seed Thickness; SW, Seed Width; SA, Seed Area; SV, Seed Volume; HSW, 100-Seed Weight.

**Figure 5:**
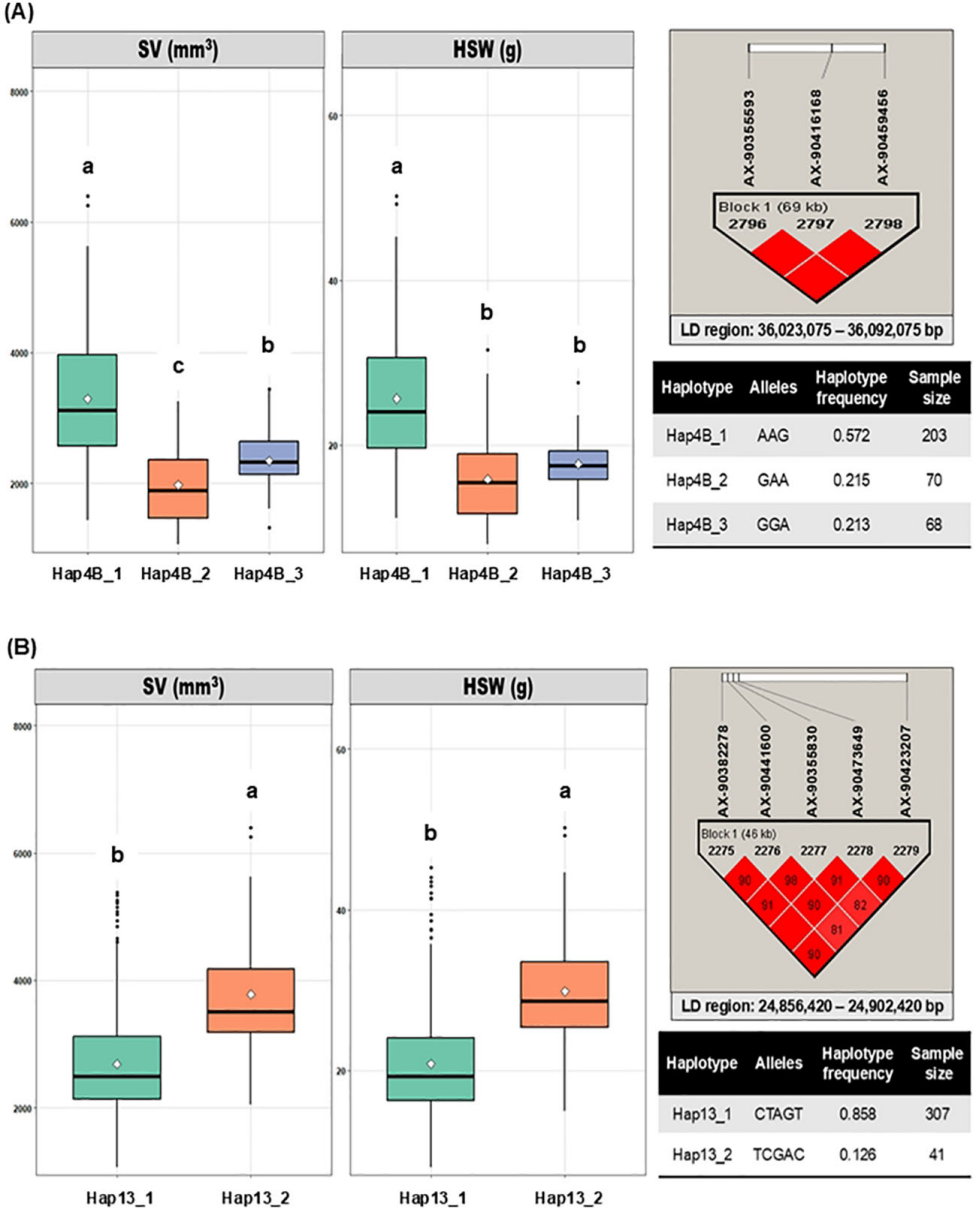
Haplotype analysis of seed size traits in the GWAS population. **(A)** Boxplots showing haplotype-trait association for seed volume (SV) and 100 seed weight (HSW) on chromosome 4. **(B)** Boxplot showing haplotype-trait association for SV and HSW on chromosome 13. The corresponding LD block, highlighted in red (r^2^ ≥ 0.8), is displayed adjacent to each boxplot. The accompanying tables summarize haplotype number, allelic combination, and haplotype frequency. Pairwise comparisons among haplotypes were conducted using Welch’s t-test (two haplotypes) and Welch’s ANOVA + Gomes-Howell at P< 0.05; different letters ‘a’, ‘b’, and ‘c’ indicate statistically significant differences while the common letters mark non-significant differences. SV, Seed Volume; HSW, 100-Seed Weight.

To further investigate the multi-allelic influence of SNP combinations on seed size, haplotype analysis was performed. The nearest adjacent SNPs surrounding each significant SNP on the same chromosome with a strong pairwise confidence interval (r^2^ ≥ 0.8) was considered a haplotype block. Only *qSS4.2* on Chr. 4 and *qSS13* on Chr. 13 showed a strong LD association with adjacent SNPs. The *qSS4.2* locus contained three haplotypes: Hap4B_1 (AAG), Hap4B_2 (GAA), and Hap4B_3 (Gabriel et al.). Similarly, *qSS13* comprised two haplotypes: Hap13_1 (CTAGT) and Hap13_2 (TCGAC), as illustrated in **Figure 5**. The haplotypes on Chr. 4 exhibited significant phenotypic variation across the accession panel: Hap4B_1 was associated with higher SV and HSW, whereas Hap4B_2 and Hap4B_3 regulated accessions with lower and intermediate values, respectively. This result indicates that the A allele at the significant SNP (AX-90355593) contributes to larger seed size, while the G allele is associated with smaller seed size. On Chr. 13, Hap13_2 exhibited a strong association with high SV and HSW, whereas Hap13_1 corresponded to lower values. Interestingly, Hap13_2, which confers large seed size, carried a higher frequency of C alleles (2^nd^ and 5^th^ position) compared with other alleles, supporting the single-SNP effect observed for *qSS13*.

### 3.5 Identification and functional annotation of candidate genes associated with significant QTLs

Within the QTL interval (± 153 kbp) for all traits, 23 genes were identified as potential candidates based on gene functional annotation, RNA-seq Atlas (Severin et al., 2010) (https://legacy.soybase.org/soyseq/) available on SoyBase (**Supplementary Figure 2**; **Supplementary Table 5**), and recent literature across different model crops. These included six genes underlying *qRP19* and sixteen genes underlying seed-specific QTLs (**Table 4**). Each QTL constituted a distinct number of genes, reflecting variation in genetic architecture across loci. Functional categorization revealed that some genes were associated with circadian rhythm, photoperiodism, and embryo or endosperm development, while others were linked to enzymatic and metabolic regulation pathways. The 16 genes within seed-specific QTLs were considered as putative regulators of seed shape and size.

**Table 4:** Candidate genes co-localized with significant QTLs and their functional annotations.

QTL	Gene	Position (bp)	Annotation	Reference
*qRP19*	*Glyma.19g221900*	47383481.47385068	AUX/IAA family	Singh and Jain, 2015
	*Glyma.19g222700*	47503589.47510481	Protein phosphatase 2C	Jung et al., 2020
	*Glyma.19g222800*	47512008.47513094	Cupin	Gabrisova et al., 2015
	*Glyma.19g223000*	47525151.47530202	Cellulase (glycosyl hydrolase family 5), mannan endo-1,4-β-mannosidase	Chen et al., 2018
	*Glyma.19g223100*	47534912.47540592	Glucose-1-phosphate adenylyl transferase large subunit 2	Chao et al., 2024
	*Glyma.19g224200*	47633059.47641958	Two-component sensor histidine kinase	Xu et al., 2013
	*Glyma.19g224300*	47645626.47647217	Cupin	Gabrisova et al., 2015
*qSS4.1*	*Glyma.04g154300*	35687483.35692338	Methyl-CpG binding domain	Frost et al., 2024
*qSS4.2*	*Glyma.04g154500*	35920206.35925176	Methyl-CpG binding domain	Frost et al., 2024
	*Glyma.04g154900*	35946734.35950623	AP2 domain	Zhang et al., 2024
	*Glyma.04g155300*	36122741.36131272	Ubiquitin	Zhang et al., 2024
*qSS13*	*Glyma.13g136000*	24857028.24860109	Adenylate kinase 1	Hsing et al., 1998
	*Glyma.13g136900*	24951190.24959983	E3 ubiquitin-protein ligase UPL4	Meng et al., 2015
*qSS20.1*	*Glyma.20g135400*	37488174.37490394	Cyclin	Hu et al., 2023
	*Glyma.20g135700*	37519433.37520110	Phytosulfokine precursor protein (PSK)	Yu et al., 2019
	*Glyma.20g136200*	37580489.37584814	Pyruvate kinase	Duan et al., 2025
	*Glyma.20g136600*	37623938.37624910	Agamous-like MADS-BOX protein AGL62	Wang et al., 2024
	*Glyma.20g136700*	37637704.37638384	MADS BOX protein	
	*Glyma.20g136800*	37646028.37647771	MADS BOX protein/AGL61	
*qSS20.2*	*Glyma.20g165200*	40267892.40271294	RING/FYVE/PHD zinc finger superfamily protein	Zhang et al., 2024
	*Glyma.20g165400*	40290011.40298274	RCC1 family with FYVE zinc finger domain	Misra and Joshi-Saha, 2023
	*Glyma.20g165800*	40349005.40358356	Serine/threonine protein kinase DRKC-related	Jiang et al., 2023
	*Glyma.20g166300*	40402142.40413612	ubiquitin interaction motif-containing protein	Zhang et al., 2024

**Figure 6:**
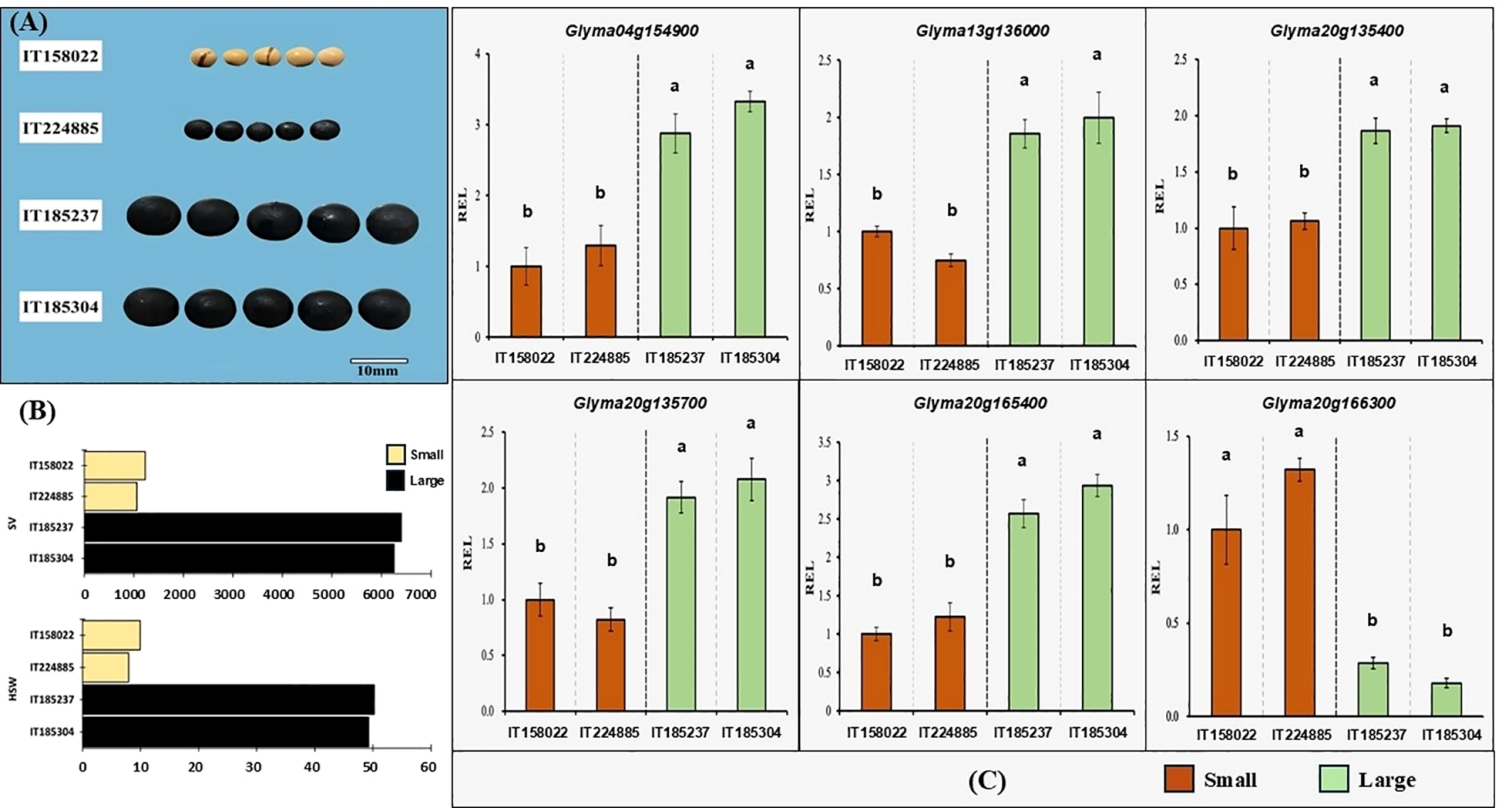
Comparative phenotypic variation and gene expression between small and large-seeded soybean accessions. **(A)** Seed size and shape of four representative accessions: IT158022 and IT224885 (small-seeded) and IT185237 and IT185304 (large-seeded). **(B)** Phenotypic differences in seed volume (SV) and 100-seed weight (HSW) at maturity. The x-axis represents accessions, and the y-axis shows trait values. **(C)** qRT-PCR analysis showing relative expression level (REL) of four candidate genes (*Glyma.04g154900*, *Glyma.13g136000*, *Glyma.20g165400*, and *Glyma.20g166300*) associated with seed specific QTLs. Brown bars represent small-seeded accessions, and green bars indicate large-seeded accessions. IT158022 was considered the control. Error bars indicate standard error. Different letters (a, b) denote statistically significant differences based on Tukey’s HSD test at P< 0.05.

To further prioritize candidate genes underlying seed size variation, downstream qRT-PCR expression analysis was focused on genes associated with the seed size related QTLs identified in this study. These putative genes were further analyzed for expression through qRT-PCR using two large-seeded and two small-seeded accessions, selected based on SV, HSW, and positive allele effects. Among them, six genes, namely *Glyma.04g154900*, *Glyma.13g136000*, *Glyma.20g135400, Glyma.20g135700, Glyma.20g165400*, and *Glyma.20g166300*, showed significant differential relative expression between the contrasting accession groups (**Figure 6**). Specifically, *Glyma.04g154900*, *Glyma.13g136000*, *Glyma.20g135400, Glyma.20g135700*, and *Glyma.20g165400* exhibited ~2 to 4-fold higher expression in large-seeded accessions compared to small-seeded accessions. In contrast, *Glyma.20g166300* showed higher expression in small-seeded accessions (IT158022 and IT224885), with ~3.45 and 5.56-fold reduced expression in the large-seeded accessions IT185237 and IT185304, respectively. Thus, these six genes were considered potential candidates for regulating seed size at the R6 stage in soybean. However, further comprehensive functional validation will be required to elucidate the precise roles of these genes in seed size regulation.

## 4 Discussion

Seed size and shape are fundamental traits directly influencing yield and commercial value in soybeans. Economically, large seeds (>20g/HSW) are suitable for cooking, oil, tofu and soymilk purposes, while small seeds (>12g/HSW) are primarily suitable for natto and bean sprout purposes (Kim et al., 2025; Zhang et al., 2025). Soybean seed parameters including SL, SW, ST, and SV collectively govern the HSW and yield. Moreover, the reproductive and seed development stages are significantly influenced by phenological traits such as DtF and DtM, which determine the seed filling phase and subsequently the seed size. The selection and development of cultivars with larger seed size, higher HSW, and early maturity are considered core strategies for enhancing productivity, thus directing the focus of soybean breeders (Bhat et al., 2025).

These quantitative traits are regulated by polygenes, making their improvement challenging through conventional breeding methods. In this perspective, molecular breeding approaches offer considerable potential for advancing soybean improvement, and their effectiveness increasingly depends on precise phenotyping. Traditional phenotyping of seed traits, which has been labor-intensive and time-consuming, is now being replaced by advanced high-throughput phenotyping platforms that provide greater efficiency and research accuracy. In particular, RGB sensor-based imaging allows multispectral phenotyping and accurate segmentation of individual seeds, providing precise spatial data for seed-related traits (Kim et al., 2025). Consequently, this study employed high-throughput phenotyping for a genome-wide association study and identified key genomic regions and candidate genes associated with seed-related traits in soybean.

### 4.1 Phenotypic variability across soybean accessions

In this study, 374 soybean accessions, including landraces from South Korea, North Korea, China, and Japan were evaluated for eight traits: two reproductive traits (DtF and DtM) and six seed size-related traits (SL, SW, ST, SA, SV, and HSW). Significant variability was observed among accessions for all traits, indicating the presence of diverse genetic resources suitable for GWAS analysis. The coefficient of variation (CV) was moderate to high (>10%) for all traits, specifically SA (23.53%), SV (35.86%), and HSW (37.25%). These traits also showed high heritability (≥ 0.9), suggesting strong potential for effective selection (**Table 1**). This outcome is consistent with previous findings (Duc et al., 2023; Niu et al., 2023).

Correlation analysis revealed strong associations among seed size traits, while reproductive traits showed weaker relationships with seed traits (**Figure 1**). These findings are supported by previous studies on phenological and seed size traits in soybean (Priyanatha et al., 2022; Jiang et al., 2023; Kim et al., 2023b). Furthermore, reports suggested a weak positive correlation (r = 0.24) between HSW and reproductive period length (days from DtF to DtM), indicating a possible independent genetic architecture for these traits (Wang et al., 2024). Remarkably, the weak correlations of DtF and DtM with seed size traits suggest minimal influence of flowering and maturity on seed development and morphology.

In contrast, the strong association of HSW with other seed size traits confirms their reliability as representative traits for seed size determination. Similar findings consistent with our study documented a strong positive association of HSW with seed size in soybean (Duc et al., 2023). This interrelationship emphasizes the utility of HSW as a key breeding target, while the corresponding assessment of related traits (SL, SW, ST, SA, and SV) can further advance selection strategies. Overall, the results highlight the complexity of trait-influencing factors while providing considerable insights for soybean breeding programs aiming to improve seed size and reproductive traits.

### 4.2 Identification of stable QTLs and haplotypes regulating reproductive and seed traits

High-throughput phenotyping-based GWAS analysis identified 99 significant SNP signals, including 40 associated with reproductive traits and 59 with seed traits. Specifically, 6 pleiotropic SNPs were identified on chromosomes 4, 13, 19, and 20 (**Table 2**). Although phenotypic data were collected across two growing seasons, genotype-by-environment (G × E) interaction analysis was not feasible due to the absence of within-year replication for reproductive traits. Consequently, GWAS was performed using pooled multi-year phenotypic observations to identify loci with consistent genetic effects across environments.

On chromosome 19, we detected a reproductive trait QTL, *qRP19* (47.39–47.69 Mbp), which overlapped with the previously reported GWAS-QTLs *R8 full maturity 4-g1* (47.59 Mbp) and *first flower 4-g81* (47.63 Mbp), associated with flowering and maturity, respectively (Sonah et al., 2015; Mao et al., 2017). Notably, *first flower 4-g81* is located at the *E3* locus, which encodes *PHYA3* (Phytochrome A type 3), a key regulator of photoperiodism, flowering, and maturity in soybean (Xu et al., 2013). This supports that *qRP19* likely functions similar to PHYA3 influencing reproductive phase duration in soybean.

On chromosome 4, two seed size QTLs, *qqSS4.1* (35.46–35.77 Mbp) and*qqSS4.2* (35.87–36.17 Mbp), were identified. These QTLs are in closeproximity and overlapped with the previously documented QTL *seed weight 7-g4* (36.37 Mbp) (Yan et al., 2017). On chromosome 13, the seed size QTL *qqSS13* lies near *seed weight 4-g13* (22.48 Mbp) (Hu et al., 2014) and *qLH13* (27.47–27.67 Mbp) regulating seed length and HSW (Luo et al., 2022). On chromosome 20, *qSS20.1* was located proximal to *qWTH20* (27.89–33.22 Mbp), known to affect ST and HSW, and coincided with *qSS20-1* (37.15–38.37 Mbp) reported to associated with SL, SW, ST and HSW (Luo et al., 2022; Wang et al., 2024). While *qSS20.2* found to be coincided with *seed weight 6-g3* at 43.66 Mbp (Sonah et al., 2015). Hence, our results found two QTLs *qSS4.1* and *qqSS13* as non-overlapping QTLs that have not been previously reported for seed size in soybean (**Supplementary Table 4**). The detection of five pleiotropic seed size QTLs overlapped or at proximal to loci previously associated with seed length, width, thickness, weight, supports the hypothesis that these regions harbor regulatory genes with coordinated effects on source-sink regulation, seed filling, seed size and multi yield components, thereby underscoring their biological relevance.

Haplotype-based analyses using multi-allelic markers provide an efficient approach to uncover rare allelic combinations and epistatic interactions across diverse accessions (Yu et al., 2024). Previous studies have reported superior haplotypes regulating seed weight (Yan et al., 2017), yield-related traits (Yang et al., 2022), flowering (Li et al., 2024), and days to flowering and maturity (Contreras-Soto et al., 2017; Perfil Ev et al., 2024) in soybean. Consistent with these findings, our study identified two haplotype blocks in *qSS4.2* and *qSS13*, comprising three and two haplotype alleles, respectively. These haplotypes were found to govern discrete phenotypic variations for SV and HSW across the accessions, offering flexibility to exploit specific haplotypes according to breeding objectives.

### 4.3 Functional insights into seed-associated candidate genes through expression analysis

The six stable pleiotropic QTLs identified through GWAS were explored for candidate genes mining within ±153kbp flanking LD region related to reproductive-linked and seed size traits (**Table 4**). Although the pleiotropic SNPs did not directly overlap with the previously characterized soybean seed size genes, their proximity to known seed regulatory genes and the presence of functionally relevant genes within the LD block suggests that these loci contribute to seed size regulation through conserved developmental and regulatory pathways.

A total of 16 putative genes were identified within the QTL regions *qSS4.1*, *qSS4.2*, *qSS13*, *qSS20.1*, and *qSS20.2* associated with seed size traits (**Table 4**). These genes represent diverse functional categories that directly or indirectly regulate seed shape, size, and weight in soybean or other model crops. For example, *Glyma.04g154300* and *Glyma.04g154500* encode methyl-CpG binding domain proteins, reported as epigenetic regulators modulating embryonic genes in *Arabidopsis* (Frost et al., 2024). Several transcription factors, including *Glyma.04g154900*, *Glyma.20g136600*, *Glyma.20g136700*, and *Glyma.20g136800*, encode AP2 and MADS-box proteins (AGL61/AGL62) implicated in circadian rhythm, floral development, and seed morphology (Wang et al., 2024; Zhang et al., 2024). Genes such as *Glyma.04g155300*, Glyma*.13g136900*, *Glyma.20g165200*, *Glyma.20g165400*, and *Glyma.20g1366300* function in ubiquitin-mediated proteasome pathways controlling seed size in soybean and chickpea (Wang and Spoel, 2022; Misra and Joshi-Saha, 2023; Zhang et al., 2025). Additional candidates, including *Glyma.13g136000* (adenylate kinase 1), *Glyma.20g135400* (cyclin), *Glyma.20g135700* (PSK), *Glyma.20g136200* (pyruvate kinase), *and Glyma.20g165800* (serine/threonine protein kinase), are linked to cell cycle regulation, enzymatic activity, and signaling pathways influencing seed size and HSW (Yu et al., 2019; Hu et al., 2023; Jiang et al., 2023; Duan et al., 2025). These putative genes highlight the complexity of hormonal and transcriptional control on biological pathways underpinning seed size determination in soybean.

The qRT-PCR validation of the 16 genes in contrasting seed-size accessions revealed six strong candidates with distinct expression patterns: *Glyma.04g154900*, *Glyma.13g136000*, *Glyma.20g135400*, *Glyma.20g135700, Glyma.20g165400*, and *Glyma.20g166300* (**Figure 6**). *Glyma.04g154900*, which encodes an AP2-domain transcription factor and is located near the QTL *Seed weight 7-g4* (Song et al., 2013; Yan et al., 2017), showed significantly higher expression in large-seeded accessions, consistent with previous reports of *GmAP2* family members positively regulating soybean HSW and seed size (Jiang et al., 2020; Zhang et al., 2024). *Glyma.13g136900*, encoding E3 ubiquitin-protein ligase UPL4, exhibited ~2-fold higher expression in large seeds, consistent with its reported role in regulating plant growth, development, and seed set through protein ubiquitination (Meng et al., 2015; Wang and Spoel, 2022). *Glyma.20g135400* and *Glyma.20g135700*, encodes for Cyclin and Phytosulfokine precursor revealed about 1.8 ~ 2.1-fold increase in expression in large seeded-accessions, respectively; similar with the previous research anticipating its regulation in seed development and seed size enhancement in soybean (Wang et al., 2024). *Glyma.20g165400*, an RCC1 family gene with a FYVE zinc finger domain, showed higher expression in large-seeded accessions. This observation supports previous reports of RCC1 proteins regulating cell division, chromosome and cytoskeleton partitioning (Misra and Joshi-Saha, 2023), as well as modulating auxin signaling pathways involved in embryogenesis and organ formation (Furutani et al., 2020). In contrast, *Glyma.20g166300*, encoding ubiquitin interaction motif (UIM)-containing protein, showed elevated expression in small-seeded accessions, consistent with the negative regulation of ovule integument expansion and organ growth by UIM-containing proteins such as DA1 (ubiquitin receptor) (Zhang et al., 2024).

Altogether, a reasonable explanation for these results is that the identified SNPs and haplotype blocks resides within the regulatory regions likely influenced seed size by modulating the expression of the nearby genes. Given that the lead SNPs are located within the LD region harboring candidate genes that shows differential expression between small-seeded and large-seeded accessions, it is probable that these variants affect cis-regulatory elements such as promoters, enhancers or transcription factor binding sites. Thus, altering gene expression and contributing to variation in seed development and size.

## 5 Conclusion

In summary, the study integrated advanced high-throughput phenotyping with GWAS to dissect the complex genetic architecture of reproductive traits (DtF and DtM) and seed size-related traits (SL, SW, ST, SA, SV, and HSW) in soybean. The GWAS analysis revealed six pleiotropic QTLs on chromosomes 4, 13, 19, and 20 that were associated with all eight traits. In addition, two haplotype blocks, Hap4B and Hap13, were found to control increased seed size phenotypes in the accessions studied. Gene mining within these stable QTLs identified six putative genes for reproductive traits and 16 for seed size traits, among which six candidate genes *viz. Glyma.04g154900*, *Glyma.13g136000*, *Glyma.20g135400, Glyma.20g135700, Glyma.20g165400*, and *Glyma.20g166300* showed distinct expression patterns in contrasting seed-size accessions. Moreover, we found that one QTL *qSS13* (previously not reported) detected through GWAS analysis, have distinct haplotype block harboring gene exhibited remarkable gene expression levels. Hence, we proposed this as a novel QTL for seed size in soybean. These results highlight the involvement of these genes in seed development, morphology, and size regulation. From applied breeding perspective, the LD defined haplotypes Hap4B and Hap13, together with their associated SNP markers, provide practical targets for marker-assisted selection. These haplotypes can be directly used to develop molecular markers for screen parental lines and breeding populations to enrich favorable alleles for seed size related traits accelerating the development of soybean cultivars. Overall, the identified loci and putative candidate genes provide novel insights into the genetic basis of seed traits and represents promising resources for molecular breeding aimed to improve soybean flowering time, maturity, seed size, and seed weight.

## Data Availability

The datasets presented in this study can be found in online repositories. The names of the repository/repositories and accession number(s) can be found in the article/**Supplementary Material**.
