# Supplementary material for: Identification of candidate loci regulating seed-associated traits in soybean using genome-wide association study and image-based high-throughput phenotyping
Source: Front Plant Sci. 2026 Mar 2;17:1727442. doi: 10.3389/fpls.2026.1727442 (PMC12989512; doi:10.3389/fpls.2026.1727442)
Supplement: Supplementary file 1 [file DataSheet1.docx]

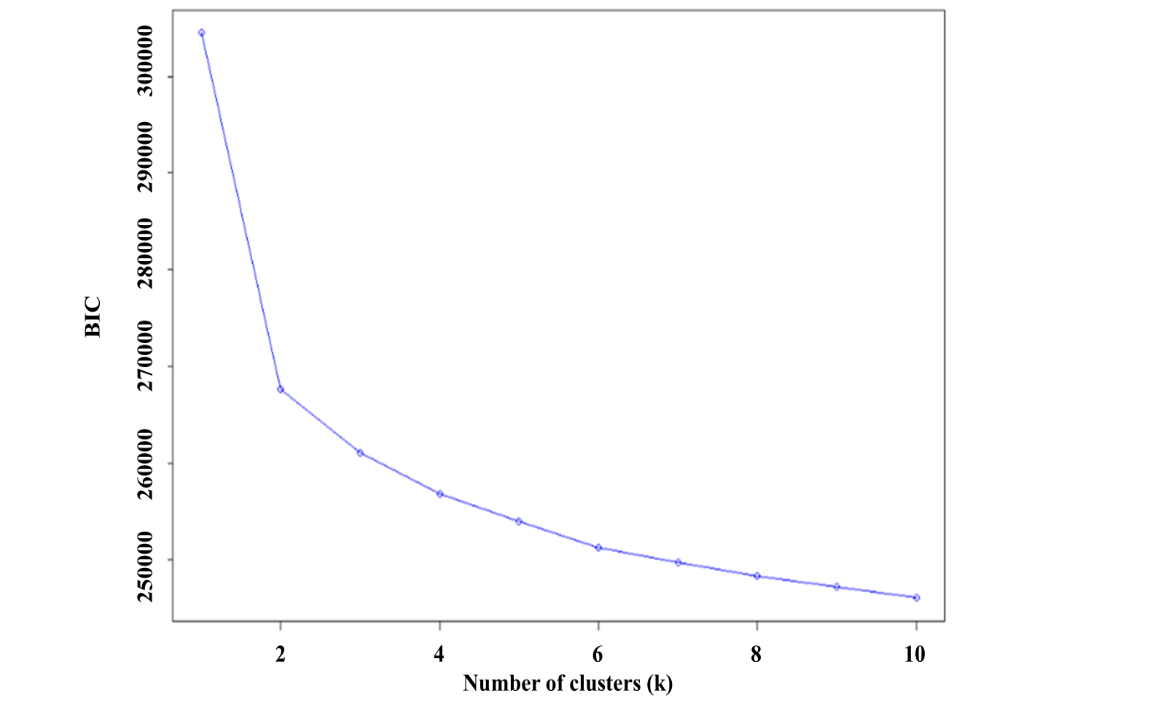


**Supplementary Figure S1.** K-means clustering plot of the 374 soybean accessions. The x-axis represents the number of clusters (k, ranging from 1 to 10), and the y-axis represents the Bayesian Information Criterion (BIC) value.


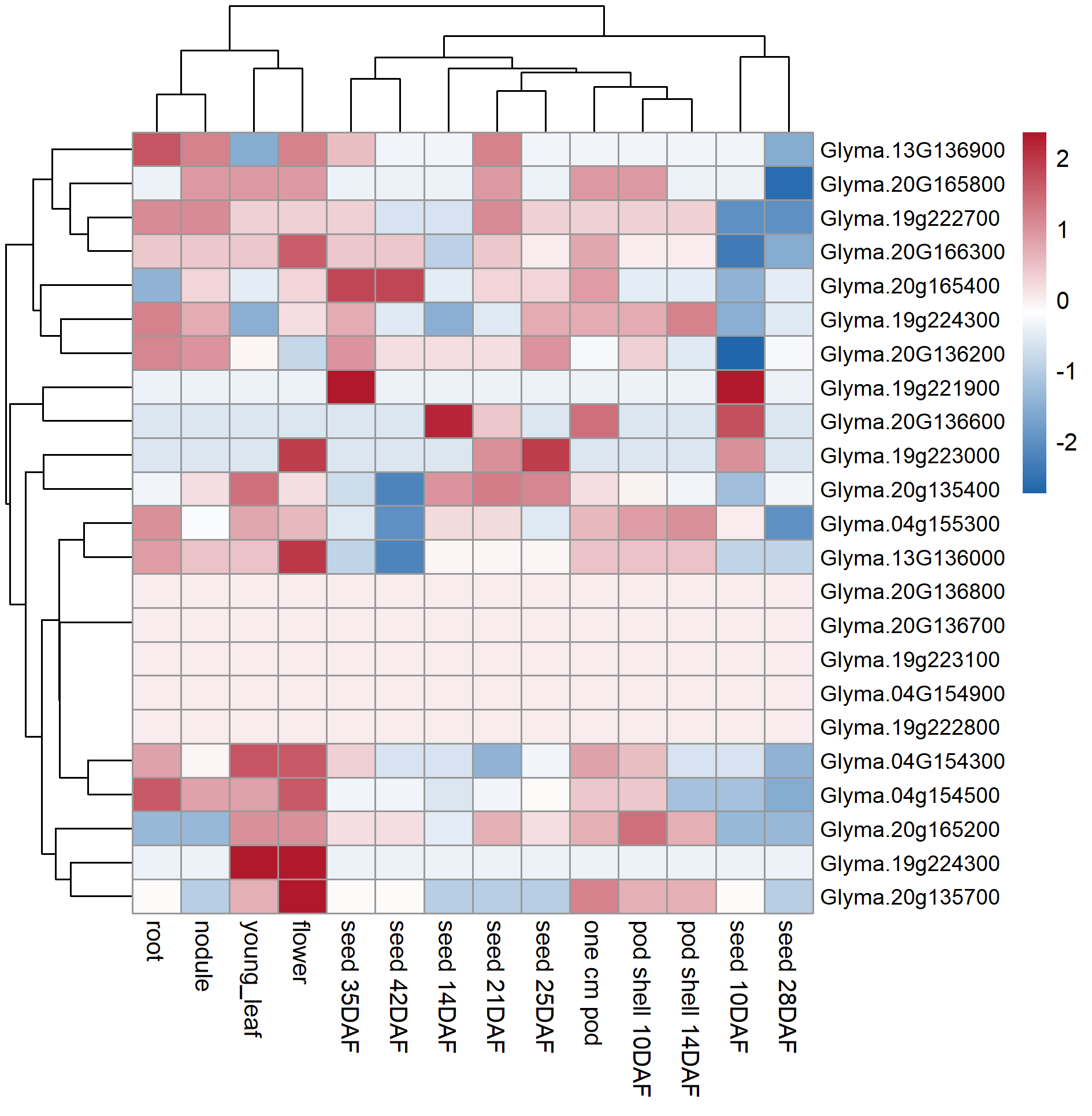


**Supplementary Figure S2.** Tissue-specific expression profiles of 23 putative genes associated with reproductive-linked and seed size traits in soybean based on RNA-seq atlas data (Severin et al., 2010) from SoyBase.
